# Multiplex Immunoassay of Plasma Cytokine Levels in Men with Alcoholism and the Relationship to Psychiatric Assessments

**DOI:** 10.3390/ijms17040472

**Published:** 2016-03-29

**Authors:** Ann M. Manzardo, Albert B. Poje, Elizabeth C. Penick, Merlin G. Butler

**Affiliations:** Departments of Psychiatry & Behavioral Sciences and Pediatrics, University of Kansas Medical Center, Kansas City, MO 64101, USA; apoje@kumc.edu (A.B.P.); epenick@kumc.edu (E.C.P.); mbutler4@kumc.edu (M.G.B.)

**Keywords:** cytokines, male alcoholism, impulsivity, anxiety, RANTES, GRO, MDC

## Abstract

Chronic alcohol use alters adaptive immunity and cytokine activity influencing immunological and hormone responses, inflammation, and wound healing. Brain cytokine disturbances may impact neurological function, mood, cognition and traits related to alcoholism including impulsiveness. We examined the relationship between plasma cytokine levels and self-rated psychiatric symptoms in 40 adult males (mean age 51 ± 6 years; range 33–58 years) with current alcohol dependence and 30 control males (mean age 48 ± 6 years; range 40–58 years) with no history of alcoholism using multiplex sandwich immunoassays with the Luminex magnetic-bead based platform. Log-transformed cytokine levels were analyzed for their relationship with the Symptom Checklist-90R (SCL-90R), Barratt Impulsivity Scales (BIS) and Alcoholism Severity Scale (ASS). Inflammatory cytokines (interferon γ-induced protein-10 (IP-10); monocyte chemoattractant protein-1 (MCP1); regulated on activation, normal T cell expressed and secreted (RANTES)) were significantly elevated in alcoholism compared to controls while bone marrow-derived hematopoietic cytokines and chemokines (granulocyte-colony stimulating factor (GCSF); soluble CD40 ligand (sCD40L); growth-related oncogene (GRO)) were significantly reduced. GRO and RANTES levels were positively correlated with BIS scales; and macrophage-derived chemokine (MDC) levels were positively correlated with SCL-90R scale scores (*p* < 0.05). Elevated inflammatory mediators in alcoholism may influence brain function leading to increased impulsiveness and/or phobia. The novel association between RANTES and GRO and impulsivity phenotype in alcoholism should be further investigated in alcoholism and psychiatric conditions with core impulsivity and anxiety phenotypes lending support for therapeutic intervention.

## 1. Introduction

Alcoholism is a complex, multifactorial behavioral disorder characterized by the loss of control over alcohol consumption often involving other drug abuse with a relapsing and remitting course leading to clinically significant impairment [1]. Direct and indirect effects of high levels of alcohol consumption have pervasive effects on multiple organ systems including the liver, gastrointestinal tract and bone, as well as brain, endocrine and other systems [2,3,4,5]. These effects are well-characterized and include alteration of cellular functioning, metabolism and energy production, impaired protein and nucleic acid synthesis, disruption of hormone regulation and function, as well as impaired absorption and transport of essential nutrients [3,4,5,6,7,8,9,10,11]. The resulting biochemical disturbances are known to trigger an enhanced inflammatory cascade involving the production of reactive oxygen species by activated macrophages and increased cytokine production by Kupffer cells in the liver and other tissues [3,4,5,12,13].

Innate immune responses involve non-specific cellular recruitment and activation of leukocytes (natural killer cells, mast cells, eosinophils and basophils) and phagocytic cells such as macrophages, neutrophils and dendritic cells for the identification and elimination of harmful pathogens [14]. Immunological responses incorporate the actions of numerous small molecules secreted by various immune cells such as cytokines, chemokines and interferons. Cytokines are 8 to 25 kilodalton polypeptides which mediate cell-cell interactions, cellular recruitment and humoral responses and include interleukins, chemokines, growth factors, interferons and colony stimulating factors [14]. Chemokines are chemoattractant proteins, which function to draw immune mediators to the site of infection. CD4^+^ T helper (Th) cells guide adaptive immune responses to specific antigens through rapid initiation of gene transcription and production of cytokines [14,15]. Increased levels of certain cytokines (T helper 1 (Th1)) generally induce an inflammatory response through activation of tumor necrosis factor α (TNFα), interleukin 1 (IL1), interleukin 6 (IL6), and interferon (IFN). T helper-2 (Th2) derived cytokines (e.g., interleukin 4 (IL4); interleukin 10 (IL10)) are considered anti-inflammatory and generally inhibit cytokine release [16].

Acute and chronic alcohol exposure interferes with cellular immune responses leading to dysregulation and functional impairment [14,15,17]. Blood cytokine levels generally decrease in response to low levels of alcohol exposure, but Bala *et al.* (2014) [18] found acute “binge” alcohol intoxication was associated with increased blood cytokine levels in healthy individuals without alcoholism [17,18]. Alcohol abuse is associated with a profound disruption of cellular recruitment and function including reduced granulocyte and lymphocyte production, bone marrow suppression and diminished bactericidal activity that significantly increases infection-related morbidity and mortality [14]. Chronic alcohol use alters monocyte-microglial activation through the activation of transcription factors (e.g., tumor necrosis factor β (TNFβ)) and induction of pro-inflammatory cytokines (e.g., TNFα, interleukin 1β (IL1β); IL6) that can precipitate inflammatory tissue injury leading to end organ failure [15,17,19]. Circulating TNFα is able to enter the brain through an active transport where it has been shown to stimulate cytokine release from endothelial cells [20,21,22]. Increased levels of monocyte chemoattractant protein-1(MCP1) and IL1β have been reported in post-mortem human brain tissue from individuals with chronic alcoholism [23,24].

Up-regulation of cellular inflammatory mediators exacerbates alcohol-related biochemical disturbances and accelerates the associated degenerative processes impacting long term outcomes. Inflammatory mediators in alcoholism precipitate organ failure (e.g., hepatitis, pancreatitis) leading to increased morbidity and mortality [3,5]. Activation/disruption of these innate immune signaling molecules in the brain are also believed to potentiate neurodegeneration and neurological dysfunction impacting mood, cognition and traits related to alcoholism including impulsiveness [2,25,26]. Circulating cytokine levels have been shown to impact mood, cognition and behavioral characteristics in psychiatric illness, including craving for alcohol [25,26,27,28,29]. Herein, we report a multiplex immunoassay to evaluate plasma cytokine disturbances in chronic severe alcoholism and the relationship of plasma cytokine levels to behavioral and psychiatric characteristics associated with alcoholism.

## 2. Results

**Figure 1:**
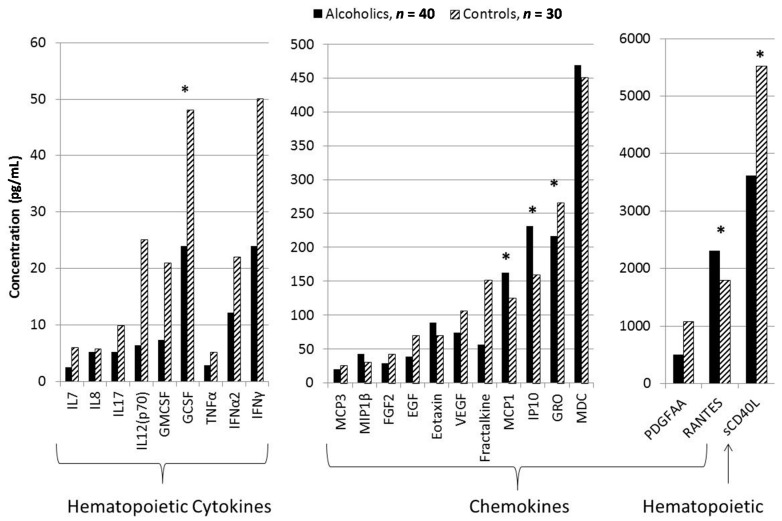
Histograms representing plasma cytokine levels for *n* = 40 adult male African-American alcoholic and *n* = 30 control participants with statistical analyses for each of the 23 detectable cytokines meeting the laboratory requirements for inclusion grouped by alcoholism diagnosis. Significance values are indicated: * *p* < 0.05 based upon generalized linear model with Bonferroni correction. Standard deviations for the alcoholism group IL7 = 2.8; IL8 = 6.3; IL17 = 10; IL12(p70) = 17; GMCSF = 19; GCSF = 12; TNFα = 4.2; interferon α2a = 12; MCP3 = 27; MIP1β = 26; FGF2 = 37; EGF = 38; eotaxin = 60; VEGF = 90; fractalkine = 186; MCP1 = 63; IP-10 (interferon γ-induced protein-10) = 365; GRO = 98; macrophage-derived chemokine (MDC) = 135; PDGFAA = 305; RANTES = 1039; soluble CD40 ligand (sCD40L) = 2933. Standard deviations for the control group IL7 = 18; IL8 = 16; IL17 = 30; IL12(p70) = 58; GMCSF = 88; GCSF = 50; TNFα = 18; interferon α2a = 51; MCP3 = 29; MIP1β = 29; FGF2 = 70; EGF = 82; eotaxin = 23; VEGF = 159; fractalkine = 608; MCP1 = 32; IP-10 = 455; GRO = 151; MDC = 165; PDGFAA = 1362; RANTES = 629; sCD40L = 3285. Plasma Cytokine Levels in African-American Men with Alcoholism Compared with Controls.

Twenty-three of the 41 (56%) cytokines tested were within a detectable and analyzable range meeting criteria for inclusion of our analyses. Plasma levels of 14 hematopoietin-derived cytokines (interleukin 1α (IL1α); interleukin 1β (IL1β); interleukin 1 receptor antagonist (IL1Ra); interleukin 2 (IL2); interleukin 3 (IL3); interleukin 4 (IL4); interleukin 5 (IL5); interleukin 6 (IL6); interleukin 9 (IL9); interleukin 10 (IL10); interleukin 13 (IL13); interleukin 15 (IL15); interleukin 12 subunit p40 (IL12(p40)); Fit3Ligand), 2 inflammatory cytokines (macrophage inflammatory protein 1 α (MIP1α); transforming growth factor α (TGFα)) and TNFβ fell below detection limits in >2/3 of subjects and were then excluded from primary analysis of cytokine levels. In addition, plasma platelet-derived growth factor subunit AB/BB (PDGFAB/BB) levels were excluded for exceeding the maximum detection limits (2191 pg/mL) in >2/3 of subjects. As expected, hematopoietic cytokines, granulocyte-colony stimulating factor (GCSF) and sCD40 L, and chemokine, growth-related oncogene (GRO), were significantly reduced in the alcohol dependent cohort (Figure 1). Conversely, inflammatory cytokines (MCP1, IP-10 (interferon γ-induced protein-10), and regulated on activation, normal T cell expressed and secreted (RANTES)) were significantly elevated in the alcohol dependent cohort. These differences were further supported by MANOVA modeling which showed a significant global effect of diagnosis (*F* = 4.53, NumDF = 23, DenDF = 44, *p* < 0.0001) on cytokine level with significance group differences in sCD40 L (*F* = 5.0, *p* < 0.03), IP-10 (*F* = 4.7, *p* < 0.03), MCP1 (*F* = 8.7, *p* < 0.004), RANTES (*F* = 4.4, *p* < 0.04), and GCSF (*F* = 14.9, *p* < 0.003) levels.

### 2.1. Psychometric Analysis in Alcohol Dependent Subjects

**Figure 2:**
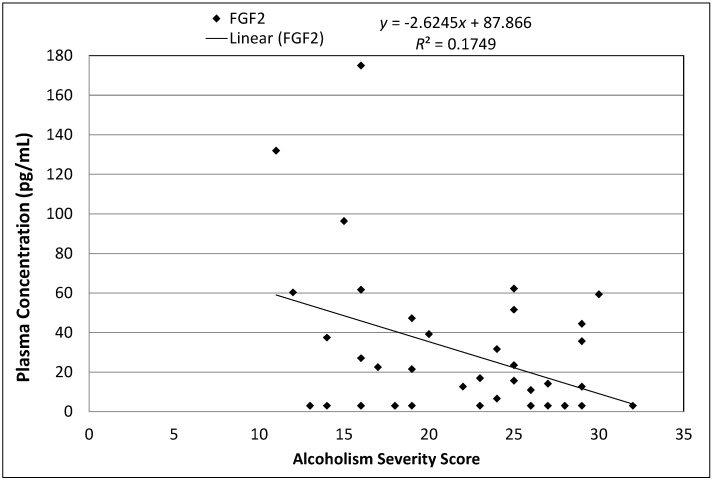
A scatter plot shows the relationship between plasma FGF2 concentration and Alcoholism Severity Score for *n* = 40 adult male African-American participants with chronic severe alcoholism. A trend line indicates the strength of the linear correlation. Hematopoietic Cytokine FGF2 Inversely Correlated with Alcoholism Severity in Men.

As anticipated due to health effects of chronic alcoholism and associated bone marrow suppression, four cytokine levels from hematopoietic (fibroblast growth factor 2 (FGF2), *r* = −0.34, *p* < 0.03; Fractalkine, *r* = −0.31, *p* < 0.05) and chemokine (monocyte chemoattractant protein 3 (MCP3), *r* = −0.31, *p* < 0.05; interleukin 12 subunit p70 (IL12p70), *r* = −0.39, *p* < 0.01) families were significantly negatively correlated with Alcoholism Severity in our sample of alcohol dependent men (Figure 2).

**Figure 3:**
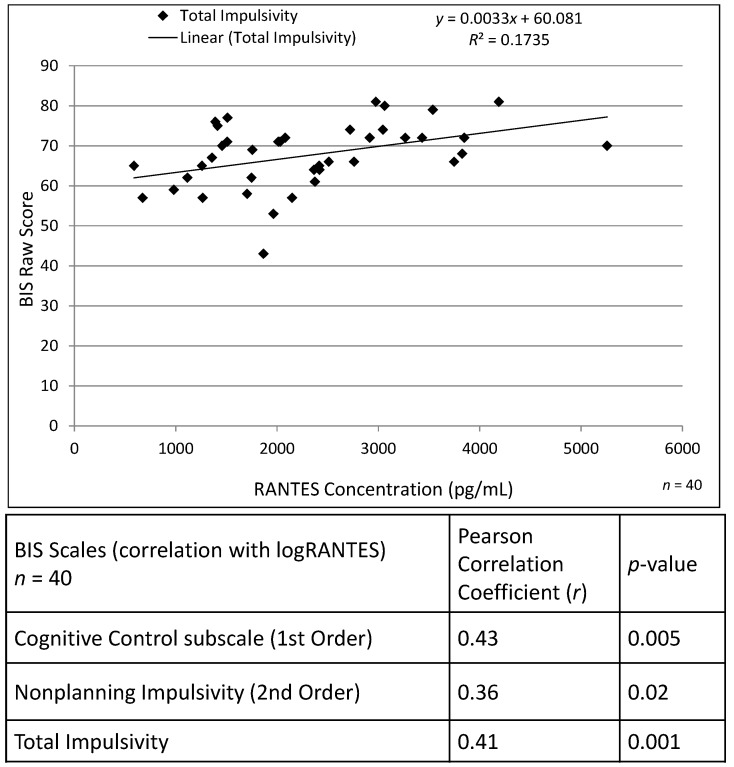
The scatter plot shows the relationship between plasma RANTES concentration and Barratt Impulsivity Raw Score for Total Impulsivity for *n* = 40 adult male African-American participants. A trendline indicates the strength of the linear correlation. The table provides Pearson correlation coefficients (*r*) between logRANTES and BIS subscales. Relationship between Plasma RANTES Levels and Barratt Impulsivity Scores in Males with Alcoholism.

**Figure 4:**
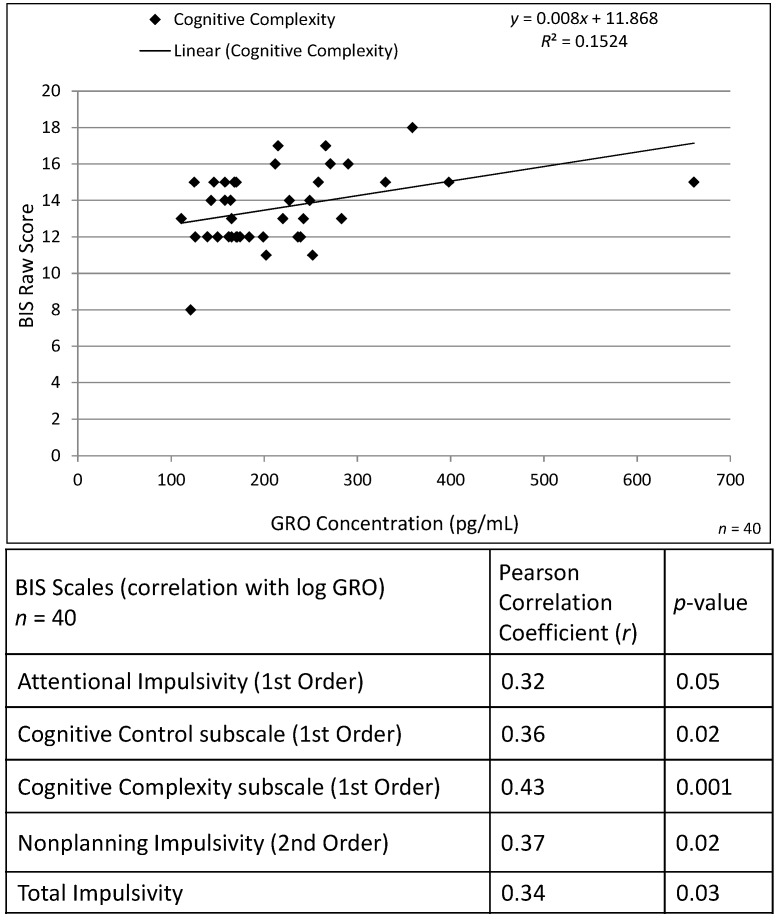
The scatter plot shows the relationship between plasma GRO concentration and Barratt Impulsivity Raw Score for Cognitive Complexity for *n* = 40 adult male African-American participants. A trendline indicates the strength of the linear correlation. The table provides Pearson correlation coefficients (*r*) between logGRO and BIS subscales. Relationship between Plasma GRO Levels and Barrett Impulsivity Scores in Males with Alcoholism.

Levels of several inflammatory cytokine were significantly positively correlated with measures from the Barratt Impulsivity Scales (BIS) and Symptom Checklist-90R (SCL-90R) psychometric assessments. As shown in Figure 3, RANTES was significantly correlated with Cognitive Control, Non-planning Impulsiveness and Total Impulsiveness on the BIS. These results remained significant after regression modeling adjusting for Alcoholism Severity Scale (ASS) (see Table 1). Levels of the chemokine, GRO, were also positively correlated with BIS measures (Attentional Impulsiveness, Cognitive Control, Cognitive Complexity, Nonplanning Impulsiveness and Total Impulsiveness; see Figure 4) despite being significantly reduced in alcoholism relative to control males. The relationship between GRO levels and Cognitive Control, Cognitive Complexity and Nonplanning Impulsiveness remained significant after regression modeling adjusted for ASS (Table 2). Epidermal growth factor (EGF) levels were positively correlated with Attentional Impulsiveness (*r* = 0.31, *p* < 0.05) and vascular endothelial growth factor (VEGF) levels were negatively associated with Perseverance (*r* = −0.33, *p* < 0.04) but these relationships were not significant after controlled regression analysis. Cytokine levels were not broadly correlated with SCL-90R scores, however, macrophage-derived chemokine (MDC, CCL22 (C-C motif chemokine 22)) levels were positively correlated with Interpersonal Sensitivity (*r* = 0.33, *p* < 0.03) and Phobia (*r* = 0.33, *p* < 0.04) subscales. The relationship between MDC levels and Phobia remained significant after adjusting for the effects of ASS (Table 3).

### 2.2. Secondary Analysis Adjusting for High IP-10 (Interferon γ-Induced Protein-10) Outliers

Mean IP-10 levels for alcohol dependent participants was 231.3 ± 365 pg/mL (50–2014 pg/mL) which was within a concentration range considered to be normal but significantly higher than control levels of 161.5 ± 455 pg/mL (64–2570 pg/mL). IP-10 is a known biomarker for viral infections, particularly hepatitis and HIV, which are increased in alcoholism. Participants with alcoholism did not report a history of hepatitis or HIV infection, but their status was not confirmed through analytical testing. To control for possible influences of active hepatitis or HIV infection on study outcomes, data were re-analyzed excluding three participants exhibiting high IP-10 levels (>800 pg/mL), which could have been attributable to active viral infection. The results of these analyses were comparable to those obtained in the initial case vs control comparisons (IP-10 levels remained significantly elevated in alcoholism) and correlations with BIS outcomes (RANTES and GRO were significantly associated with impulsiveness). However, correlations between cytokine level and ASS were weakened and no longer considered significant for Fractalkine, IL17 and MCP3. Interestingly, correlational analysis of MDC levels showed enhanced association and positive correlations with multiple SCL-90R subscales including: somatization (*r* = 0.42, *p* < 0.01); obsessive symptoms (OCD (*r* = 0.36, *p* < 0.03)); interpersonal sensitivity (*r* = 0.43, *p* < 0.001); depression (*r* = 0.34, *p* < 0.05); anxiety (*r* = 0.37, *p* < 0.02); phobia (*r* = 0.40, *p* < 0.01); GSI (*r* = 0.38, *p* < 0.02). MCP3 levels were also inversely correlated with OCD scores (*r* = −0.33, *p* < 0.04).

## 3. Discussion

Plasma cytokine disturbances were identified in our population of African-American males with chronic severe alcoholism relative to African-American control males of a similar age range. Suppression of GCSF and sCD40L impacting hematopoietic stem cell lineages and chemokine, GRO, were associated with chronic alcoholism. GCSF is a cytokine hormone that stimulates bone marrow to produce granulocytes and stem cells for the survival, proliferation, differentiation, and function of neutrophil precursors and mature neutrophils. Soluble CD40 ligand (sCD40L) is found in platelets and is a biomarker of platelet activation and the coagulation cascade. GRO is produced by macrophages, neutrophils and epithelial cells and has been shown to impact the migration of oligodendrocyte precursors in spinal cord development and angiogenesis [30,31,32]. The levels of several hematopoietic cytokines were also negatively correlated with lifetime severity of alcoholism, including IL12(p70), FGF2, fractalkine, as well as chemokine, MCP3, required for activation of immune signaling, leukocyte and monocyte response and wound healing. These disturbances and associations likely reflected generalized suppression of bone marrow and immune mediators resulting from alcohol toxicity and chronic severe alcoholic illness. These results are consistent with previous literature that indicates complex and damaging effects of alcoholism on the body impacting immune function.

Inflammatory mediators MCP1 (CCL2), RANTES (CCL5) and IP-10 were found to be elevated in alcoholism, which has been reported previously in association with alcoholism-related viral infection, alcoholic liver and pulmonary disease [33,34,35]. These inflammatory mediators are often secreted in response to γ-interferon (IFNγ), which was not elevated in our sample through a Th1 mediated response to cellular pathogens [33,34,35]. MCP1 regulates migration and infiltration of monocytes and macrophages during inflammatory processes and dysregulation of MCP1 signaling has been implicated in the pathological processes of various disease states particularly cardiovascular disease [36]. MCP1 was induced by alcohol exposure in animal models of alcoholism and elevated in post-mortem brain samples from alcohol dependent individuals [23,24]. Our results further support MCP1 activation in alcoholism and a potential role for dysregulation of MCP1 signaling in alcoholism pathology, but we failed to identify any significant association between MCP1 levels and depression or other psychiatric symptomatology in our chronically alcoholic sample of African-American males as previously observed in non alcohol dependent individuals [26]. However, MDC elevations were associated with Interpersonal Sensitivity and Phobia symptomatology, which may be related to moderating factors involved in alcohol consumption as well as psychiatric co-morbidity of patients with alcohol dependence. MDC is a constitutively active chemokine augmented by IL-1 and TNF and may also possess anti-HIV activity [37].

In our study, plasma concentrations of RANTES were significantly elevated in alcoholism and positively correlated with empirical measures of impulsiveness. RANTES promotes inflammation through the recruitment of T cells, eosinophils, basophils and the activation of natural-killer cells. RANTES activation has been previously implicated in pathological processes for several inflammatory and behavioral disorders including atherosclerosis, autoimmune disorders (e.g., asthma, lupus erythematosus), depression, traumatic brain injury, schizophrenia and obesity [26,38]. To our knowledge, this is the first report of an association between plasma RANTES levels and impulsiveness or alcoholism. Up-regulation of RANTES and associated cellular inflammatory mediators may exacerbate alcohol-related biochemical disturbances and accelerate neurodegenerative processes impacting behavior either through direct cytotoxic effects of cytokine mediators or secondary to other biochemical disturbances impacting cellular functioning or energy production [26]. The precise biochemical mechanisms involved in these processes require characterization. Racial, ethnic and gender variations in RANTES levels have also been documented with Caucasians displaying higher levels than African-Americans and females displaying higher levels than males. The plasma RANTES concentration ranges observed in our sample (2313 ± 1039 pg/mL, range 588–5258 in alcoholism; 1790 ± 629 pg/mL, range 830–3331 in controls) corresponded with levels reported for African-American males with hypertension (mean = 2772 pg/mL; median = 1543 pg/mL) [39]. It is not known how racial, ethnic and gender variation in circulating RANTES levels impacts associations with behavior such as impulsiveness, but future studies should incorporate appropriate controls for these influences.

The chemokine, GRO, was reduced in alcoholism but positively correlated with impulsivity measures in our subjects. Cytotoxic effects of GRO activation in childhood or adolescence may influence neurodevelopmental processes leading to impulsiveness and hence increased vulnerability to the development of alcoholism. Inter-correlation between inflammatory mediators and impulsiveness may represent novel biomarkers of value for the prediction of alcoholism vulnerability, course and responsiveness to recovery. Cytokine levels were not broadly correlated with psychiatric symptomatology as assessed by the SCL-90R instruments. However, MDC was positively correlated with several measures of anxiety and phobia, which may be evidence of specific neurodegenerative or inflammatory processes. MDC, RANTES, and MIP-1α/β all target the CCR4 receptor which activates phosphatidylinositol-calcium second messenger and Akt signaling pathways in endothelial cells mediating angiogenesis or angiostasis with activity in the CNS. Alterations in vascular growth, maintenance and density may also influence frontal-temporal development and function impacting mood, cognition and impulsiveness. The MDC gene (*CCL22*) is also homologous to the RANTES gene (*CCL5*) [37], but did not impact impulsivity measures suggesting the possible involvement of different physiological processes.

The multiplex immunoassay methodology utilized did not detect very low circulating levels of many cytokines, particularly those in the interleukin family, limiting our assessments of several important targets including (e.g., IL1β, IL2 and IL6) previously associated with psychiatric symptomatology and alcoholism [14,15,25,29]. No additional confirmatory testing was performed to verify the immunoassay results. The drinking status of control participants was determined by self-report rather than direct assessment, which may have contributed to variance in measured cytokine levels obscuring study findings. Participant blood samples were not tested for the presence of active hepatitis, HIV infection or liver disease, which could have significantly impacted psychometric assessments and immunological response, but the sample was typical of the substance abuse population with evidence of compromised mental and physical health. Further, secondary analyses excluding three participants with high plasma levels of IP-10 (a biomarker for viral infection) did not substantially alter study findings.

## 4. Materials and Methods

### 4.1. Subjects

Forty adult male African-American participants (mean age 51 ± 6 years; range 33–58 years) who met DSM-IV-TR criteria for alcohol use disorder were enrolled for a clinical trial on alcoholism with oversight of Kansas University Medical Center (KUMC) Human Subjects Research Committee [40,41]. The majority of participants (92%) reported a family history of alcoholism and 70% reported previous history of substance use/abuse. Thirty African-American males (mean age 48 ± 6 years; range 40–58 years) with no self-reported history of alcohol problems were included as controls. Peripheral blood samples from control males were obtained commercially from an established blood collection unit (Golden West Biologicals, Inc., Memphis, TN, USA). No psychiatric measures were available for the control subjects.

### 4.2. Study Instruments and Procedures for Males with an Alcohol Use Disorder

A comprehensive interview was completed on each participant by a trained research nurse to determine final eligibility based upon DSM-IV-TR diagnostic criteria for an alcohol use disorder as previously reported [40,41]. The interview included the lifetime Alcohol Severity Scale (ASS) that was designed to reliably review all major social, clinical and physical sequelae associated with alcohol abuse and dependence [40,42,43,44]. The sum of this 33-item scale represents a reliable measure of lifetime alcoholism severity. The Barratt Impulsivity Scale (BIS) was administered to measure various dimensions of impulsiveness. The BIS is a 30-item questionnaire with subject rated responses (Rarely/Never, Occasionally, Often, Almost Always/Always) that are scored to yield a total score, six first-order factor scores (attention, motor, self-control, cognitive complexity, perseverance and cognitive instability) and three second-order factor scores (attentional, motor, and non-planning impulsiveness) [45]. The attentional impulsiveness factor scale is designed to assess task-focus abilities, the level of intrusive and racing thoughts. The motor impulsiveness factor scale is designed to assess consistency of lifestyle and the tendency to act on the spur of the moment and the non-planning impulsiveness factor scale is designed to assess care associated with thinking and planning and enjoyment of challenging mental tasks. The Symptom Checklist 90-R (SCL-90-R) is a measure of current psychiatric symptomatology over the past week [46]. The SCL-90 consists of 90 self-report items rated according to severity with a score of 1 to 4 that produces 9 primary factor-based symptom dimensions (somatization, obsessive-compulsive, interpersonal sensitivity, depression, anxiety, hostility, phobic anxiety, paranoid ideation and psychoticism) designed to provide information on specific areas of psychological distress and one general dimension of psychiatric severity (Global Severity Index (GSI)) summarizing overall level of psychological distress.

### 4.3. Cytokine Assay and Analysis

Plasma cytokine levels were determined using multiplex sandwich immunoassays with the Milliplex Human 41 Cytokine/Chemokine Premixed Kit (Millipore; Billerica, MA, USA) according to manufacturer’s protocol. The 41 cytokines included in the kit belong to four families: hematopoietins, chemokines, growth factors and interferons and included: hematopoietin (IL1α; IL1β; IL1Ra; IL2; IL3; IL4; IL5; IL6; IL7; IL9; IL10; IL12p40; IL12p70; IL13; IL15; IL17; sCD40L; Flt3 ligand; GCSF; and GMCSF); chemokines (EGF; Eotaxin; FGF2; Fractalkine; RANTES; GRO; IL8; IP-10; MCP1; MCP3; MDC; MIP1α; MIP1β; TGFα; and VEGF); platelet derived growth factors (PDGFAA and PDGFAB/BB); tumor necrosis factors (TNFα and TNFβ) and interferons (IFNα2 and IFNγ).

Blood plasma (25 µL) and a concentration standard, or a Milliplex quality control standard were combined with pre-mixed antibody-coupled fluorescent beads and assay buffer followed by overnight incubation at 4 °C. All incubation steps were carried out on a micro-titer plate shaker at 300 rpm. The following day, the samples were washed, followed by incubation with secondary detection antibodies for 1 h at room temperature (RT). The samples were then put through another series of washes followed by addition of the Streptavidin-Phycoerythrin detection solution. This mixture was incubated for 30 min at RT. Each sample was run in duplicate. Following incubation, sheath fluid was added to each sample well and the plate was read on the Luminex 200TM instrument (Luminex Molecular Diagnostic; Toronto, ON, Canada) based on fluorescent-bead technology. The following cytokines/chemokines were analyzed using the Luminex 200TM v2.3 software (Luminex Corporation, Austin, TX, USA)with the indicated minimum detectable concentration levels given in pg/mL values: IL1α (0.4), IL1β (0.4), IL1Ra (0.4), IL2 (0.4), IL3 (0.4), IL4 (0.9), IL5 (0.4), IL6 (0.4), IL7 (1.1), IL9 (0.4), IL10 (0.4), IL12p40 (2.9), IL12p70 (2.0), IL13 (1.5), IL15 (1.6), IL17 (0.1), CD40L (5.1), Flt3 ligand (2.8), GCSF (1.0), GMCSF (1.1), EGF (2.8), Eotaxin (2.2), FGF2 (6.0), Fractalkine (5.0), RANTES (1.2); GRO (9.9), IL8 (0.4), IP-10 (8.6), MCP1 (1.9), MCP3 (1.3), MDC (3.6), MIP1α (1.4), MIP1β (1.4), TGFα (0.4), VEGF (0.3), TNFα (0.4), TNFβ (2.7), IFNα2 (3.1), IFNγ (0.9), PDGFAA (0.4) and PDGFAB/BB (2.2). Plasma cytokine concentraptions were calculated using a standard curve derived from the reference cytokine concentration standards supplied by the manufacturer. The inter-assay coefficient of variation for the cytokines ranged from 3.7% to 17.2% while the intra-assay coefficient of variation ranged from 4.5% to 13.8%. Plasma samples were analyzed using numbers and blinded to alcoholism status during each assay run.

### 4.4. Statistical Analysis

The final analyses considered data that fell within the detections limits of the Luminex assay. Data falling below the detection limit were replaced with values one half of the minimum detection level for that cytokine as reported in previous studies [47,48,49]. Cytokines with >2/3 of values outside the detectable range were excluded. Log-transformed data were analyzed and descriptive statistics (mean, std dev) were generated. Log-transformed data met the necessary statistical criteria for the assumption of normality showing equal variance and a near linear residual plot. Primary analyses compared group means for log-transformed cytokine levels using generalized linear regression modeling with Bonferroni correction, multi-variate analysis of variance and correlations with the Pearson correlation coefficient. Findings with *p*-values of <0.05 were considered significant. All analyses were carried out using SAS version 9.2 (SAS Inc., Cary, NC, USA).

## 5. Conclusions

Cytokine biology in chronic severe alcoholism is complex with many interacting influences related to race and sex, the general health of the individual and lifetime severity of alcoholism. Up-regulation of cellular inflammatory mediators may exacerbate alcohol-related biochemical disturbances, disrupt frontal cortical angiogenesis and neurodevelopment or accelerate neurodegenerative processes impacting behavior either through direct cytotoxic effects of cytokine mediators or secondary to other biochemical disturbances impacting cellular functioning or energy production. Further studies are needed to confirm these relationships and characterize the biochemical mechanisms involved. Inter-correlations between inflammatory mediators and psychometric measures of distress and impulsiveness may represent novel biomarkers of value for consideration in future studies of alcoholism course and responsiveness to recovery. The role of RANTES, GRO and MDC should be further investigated in alcoholism and other psychiatric conditions with core impulsivity and anxiety phenotypes and address therapeutic interventions when needed.

## Figures and Tables

**Table 1:** Linear Regression model of logRANTES (regulated on activation, normal T cell expressed and secreted) on Barratt Impulsivity Scales (BIS) outcomes.

Main Effects	*F* Value	Degrees of Freedom	*p*-Value
*Cognitive Control*			
logRANTES	9.2	1	**0.004**
Alcoholism Severity Score	1.1	1	0.30
logRANTES × ASS	2.3		0.14
Overall Model (*R*^2^ = 0.26)	4.2	3 (36 error)	**0.01**
*Nonplanning Impulsivity*			
logRANTES	5.7	1	0.02
Alcoholism Severity Score	0.19	1	0.67
Overall Model (*R*^2^ = 0.14)	2.9	2 (37 error)	0.06
*Total Impulsivity*			
logRANTES	7.4	1	**0.01**
Alcoholism Severity Score	0.12	1	0.73
Overall Model (*R*^2^ = 0.17)	3.7	2 (37 error)	**0.03**

Linear regression model of logRANTES levels on BIS subscales. Bold numbers indicate statistical significance at *p* < 0.05.

**Table 2:** Linear regression modeling of logGRO (growth-related oncogene) on BIS outcomes.

Main Effects	*F* Value	Degrees of Freedom	*p*-Value
*Cognitive Control*			
logGRO	5.9	1	**0.02**
Alcoholism Severity Score	2.6	1	0.12
logGRO × ASS	0.39	1	0.54
Overall Model (*R*^2^ = 0.20)	2.9	3 (36 error)	**0.05**
*Cognitive Complexity*			
logGRO	8.4	1	**0.01**
Alcoholism Severity Score	0.34	1	0.56
logGRO × ASS	0.14		0.71
Overall Model (*R*^2^ = 0.20)	2.9	2 (36 error)	**0.05**
*Nonplanning Impulsivity*			
logGRO	6.2	1	**0.02**
Alcoholism Severity Score	1.0	1	0.32
Overall Model (*R*^2^ = 0.16)	3.6	2 (37 error)	**0.04**

Bold numbers indicate statistical significance at *p* < 0.05.

**Table 3:** Linear Regression Model of logMDC (macrophage-derived chemokine) on SCL-90-R Phobia Score.

Main Effects	*F* Value	Degrees of Freedom	*p*-Value
logMDC	5.0	1	**0.03**
Alcoholism Severity Score	3.2	1	0.08
Overall Model (*R*^2^ = 0.18)	4.1	2 (37 error)	**0.02**
*Interaction Model*			
logMDC	4.9	1	**0.03**
Alcoholism Severity Score	3.1	1	0.08
logMDC × ASS	0.12	1	0.73
Overall Model (*R*^2^ = 0.18)	2.7	3 (36 error)	0.06

Bold numbers indicate statistical significance at *p* < 0.05.

## Abbreviations

ASS	Alcoholism Severity Scale
BIS	Barratt Impulsivity Scales
EGF	Epidermal growth factor
FGF	Fibroblast growth factor
GCSF	Granulocyte-colony stimulating factor
GRO	Growth-related oncogene
HIV	Human immunodeficiency virus
IFN	Interferon
IL	Interleukin
IP-10	Interferon γ-induced protein 10
MDC	Macrophage-derived chemokine (also CCL22)
MCP	Monocyte chemoattractant protein
MANOVA	Multivariate analysis of variance
OCD	Obsessive compulsive disorder
SCL-90R	Symptom Checklist-90R
sCD40L	Soluble CD40 ligand
RANTES	Regulated on activation normal T cell expressed and secreted
Th	T helper
TGF	transforming growth factor
TNF	Tumor necrosis factor
VEGF	Vascular endothelial growth factor
